# Cardiac findings in infants with in utero exposure to Zika virus- a cross sectional study

**DOI:** 10.1371/journal.pntd.0006362

**Published:** 2018-03-26

**Authors:** Dulce H. G. Orofino, Sonia R. L. Passos, Raquel V. C. de Oliveira, Carla Verona B. Farias, Maria de Fatima M. P. Leite, Sheila M. Pone, Marcos V. da S. Pone, Helena A. R. Teixeira Mendes, Maria Elizabeth L. Moreira, Karin Nielsen-Saines

**Affiliations:** 1 Department of Pediatrics, Fernandes Figueira Institute, Oswaldo Cruz Foundation, Rio de Janeiro, Brazil; 2 Souza Marques School of Medicine, Rio de Janeiro, Brazil; 3 Laboratory of Clinical Epidemiology, Evandro Chagas National Institute of Infectology, Oswaldo Cruz Foundation, Rio de Janeiro, Brazil; 4 Department of Infectious Diseases, Fernandes Figueira Institute, Oswaldo Cruz Foundation, Rio de Janeiro, Brazil; 5 Department of Clinical Research, Fernandes Figueira Institute, Oswaldo Cruz Foundation, Rio de Janeiro, Brazil; 6 Department of Medicine, Division of Infectious Diseases, University of California, Los Angeles, California, United States of America; Instituto de Ciências Biológicas, Universidade Federal de Minas Gerais, BRAZIL

## Abstract

**Background:**

Antenatal exposure to Zika virus (ZIKV) is related to severe neurological manifestations. A previous study in Brazil reported an increased incidence of non-severe congenital heart defects in infants with diagnosis of congenital Zika syndrome but without laboratory confirmation of ZIKV infection in the mother or infant. The objective of this study is to report echocardiographic (ECHO) findings in infants with laboratory confirmed antenatal exposure to ZIKV.

**Methodology:**

Cross sectional study of cardiologic assessments of infants born between November 2015 and January 2017 with confirmed vertical exposure to ZIKV in Rio de Janeiro, Brazil.

**Results:**

The study enrolled 120 children with a median age of 97 days (1 to 376 days). In utero exposure to ZIKV was confirmed in 97 children (80,8%) through positive maternal polymerase chain reaction (PCR) results during pregnancy or a positive PCR result at birth; 23 additional children (19.2%) had maternal positive PCR results during pregnancy and postnatally. Forty- eight infants (40%) had cardiac defects noted on ECHO. Thirteen infants (10.8%) had major cardiac defects (atrial septal defect, ventricular septal defect, patent ductus arteriosus). None of the defects were severe. The frequency of major defects was higher in infants whose mothers had a rash in the 2nd trimester of pregnancy, or who had altered Central Nervous System (CNS) imaging postnatally or were preterm.

**Conclusions:**

Infants with *in utero* ZIKV exposure have a higher prevalence of major cardiac defects, however none were severe enough to require immediate intervention. For this reason, guidelines for performance of postnatal ECHO in this population should follow general newborn screening guidelines, which significantly reduces the burden of performing emergent fetal or neonatal ECHOs in a setting where resources are not available, such as most Brazilian municipalities.

## Author summary

We performed cardiologic assessment of 120 infants born between November 2015 and January 2017 with confirmed vertical exposure to Zika virus (ZIKV). The diagnosis of ZIKV exposure was confirmed by PCR in maternal blood specimens and/or amniotic fluid during pregnancy and/or infant specimens including PCR of the cerebrospinal fluid (CSF) and/or urine following birth. Our study found a 10.8% incidence of structural heart defects in infants with a history of intrauterine exposure to ZIKV, a rate considerably higher than that observed in the general population. However, no patients were found to have severe structural heart defects. The presence of major defects was increased in infants whose mothers had a history of rash in the 2nd trimester, infants with altered CNS imaging and/or preterm infants. As severe heart defects requiring immediate intervention were not identified, guidelines for performance of postnatal ECHO in this population should follow the same criteria as the ones applicable to newborns in general, particularly since resources for performance of fetal and neonatal echocardiograms are not routinely available in most Brazilian municipalities

## Introduction

Zika virus (ZIKV) is an arbovirus of the Flavivirus genus like Dengue virus (DENV) and Yellow Fever virus (YFV) [1]. In response to the search for the causative agent of a “dengue-like” exanthematous disease in 2015, ZIKV was identified for the first time in Brazil by reverse transcriptase polymerase chain reaction (RT- PCR) in blood samples from patients in the Northeast region of the country [1]. Autochthonous transmission of ZIKV was confirmed in Brazil months later [2]. That same year, an increase in the number of infants with microcephaly born months after the outbreak was observed [3], thereby corroborating the possibility of congenital defects caused by vertical transmission of ZIKV. In 2016 the virus was identified by RT-PCR in tissue samples from two neonates with severe microcephaly [4] and by the detection of ZIKV in the amniotic fluid from two fetuses of women with history of rash during pregnancy [5,6,7]. Diagnosis of vertical exposure to ZIKV is therefore based on positive RT-PCR in mothers with suspected symptoms during pregnancy [5]. Congenital Zika syndrome, resulting from vertical transmission of the virus, includes microcephaly, specific findings from imaging of the central nervous system, visual and auditory deficits, and arthrogryposis [8,9,10].

Just as there are no reports, prior to 2015, of severe neurological manifestations related to vertical transmission of any Flavivirus, there are no reports of congenital heart defects in patients with confirmed vertical exposure to these viruses. A study in 2015 in Pernambuco State, Brazil, with 103 patients with diagnosis of congenital Zika syndrome but without laboratory confirmation of ZIKV infection in mothers or infants showed an increased incidence of non-severe congenital heart defects [11].

With the increase in the number of studies ordered in mother-infant pairs with suspected or confirmed ZIKV infection in pregnancy, it becomes important to verify which tests are really necessary to promote better use of health resources in Brazil and elsewhere while still enabling optimal care for these patients [12]. During the ZIKV epidemic in Rio de Janeiro, we performed 2D echocardiogram in neonates and infants with suspected *in utero* exposure to the virus. The present study describes the cardiac findings identified in this population.

## Methods

We performed a cross-sectional study of infants born between November 2015 and January 2017 who had confirmed vertical exposure to ZIKV and were followed at our outpatient Pediatric Infectious Disease Clinic at the Fernandes Figueira Institute (IFF-FIOCRUZ). The infants who received echocardiograms were born either at IFF or other institutions with positive ZIKV maternal PCR results during pregnancy or positive PCR results at birth. Our institution is a national research referral center for high risk pregnancies and high risk infants. For this reason, during the Zika epidemic we were the major referral center in Rio de Janeiro for cases of suspected maternal or infant ZIKV infection.

All infants had negative serology result for other congenital infectious including toxoplasmosis, rubella, cytomegalovirus, hepatitis B, C and HIV, in addition to no diagnosis of genetic syndromes potentially accompanied by congenital heart defects. None of the children had previous fetal ECHOs. The diagnosis of ZIKV exposure was confirmed by RT-PCR in a blood sample from the mother and/or in the amniotic fluid during pregnancy and/or in infant specimens including PCR of the cerebrospinal fluid (CSF) and/or urine following birth.

The following data were abstracted from medical records: maternal history of rash during pregnancy, gestational age at birth, diagnosis of congenital Zika syndrome, results of infants CNS imaging, and PCR results of maternal and infant specimens.

Minor ECHO defects were defined as: persistent foramen ovale (PFO), patent ductus arteriosus (PDA) in premature infants up to three months of age or in term infants up to 15 days of age, physiological tricuspid regurgitation, and physiological pulmonary branch stenosis in premature infants. All other ECHO defects were considered major structural defects. Congenital heart defects that required any therapeutic intervention in the first days of life were considered severe structural heart defects [13].

Infant cardiologic evaluation included a complete clinical examination and full 2D and M-mode transthoracic echocardiography with pulsed and continuous Doppler and color Doppler, performed by pediatric cardiologists from the Pediatric Cardiology Department of IFF. The equipment used was the Siemens Acuson X300 Echocardiography system, and the studies were performed without sedation. The cardiologic evaluation was performed between 1 and 376 days of age.

Data analysis was descriptive and used absolute and relative frequencies of qualitative variables, like gender, PCR results and major ECHOs defects. Additionally, the 95% confidence interval (95% CI) of relative frequencies was provided. Age in days was described by the median, minimum and maximum. The association between qualitative variables and major ECHO defects (presence or absence) was based on the comparison of absolute and relative frequencies, considering differences of at least 10 percentage points (pp) as indicative of a potential difference between patients with and without major ECHO alterations. In addition, individual data were provided on all patients with major ECHO defects. Due to the study’s descriptive design and the small number of major ECHO defects, we avoid using statistical testing and we added Fisher exact test as only an additional information. The R software version 3.3.3 and the package epiDisplay were used to perform the data analyses.

The study was approved by the Institutional Review Board of the Brazilian National Institute of Infectious Diseases (INI-FIOCRUZ). All parents or guardians provided written informed consent. All data analyzed were anonymized.

## Results

Among 186 children who met inclusion criteria (maternal and/ or infant PCR results confirming in utero ZIKV exposure) 120 were brought by their parents or guardians to the pediatric clinic for a cardiac consult and performance of an echocardiogram. Sixty-six children were excluded from the study because they did not come for an echo study. The median age among the 120 participants was 97 days (1 to 376 days) with equal distribution of boys and girls. Eigthy nine subjects (74.2%; 95%CI = 65.4–81.7) were born at term. Ninety-seven infants (80.8%; 95%CI = 72.6–87.4) had confirmation of *in utero* exposure to ZIKV by a positive maternal PCR during pregnancy or a positive PCR result at birth. A total of 23 additional children (19.2%; 95%CI = 12.6–27.6) had positive maternal PCR results during pregnancy and a positive PCR result at birth. A total of 84 infants in the cohort had microcephaly and 25 had the diagnosis of vertical exposure to ZIKV because microcephaly was diagnosed on the morphologic ultrasound during pregnancy or were born with congenital Zika syndrome.

Forty-eight infants (40%; 95%CI = 31.2–49.3) had cardiac defects noted on ECHO. A PFO was the most common finding responsible for 72.9% (35 patients, 95%CI = 58.2–84.7) of ECHO abnormalities. Infants with a PFO had a median age of 29 days, ranging from 2 to 372 days. PDA was found in 6 patients (12.5%, 95%CI = 4.7–25.2). The median age of infants with PDA was 98 days, ranging from 2 to 288 days. Ventricular septal defects (VSDs) were seen in 4 patients (4.2%, 95%CI = 0.5–14.3) all of them were small perimembranous VSDs. Atrial septal defects (ASDs) of the small *ostium secundum* type were present in three patients (6.3%, 95%CI = 0.1–17.2). Physiological tricuspid regurgitation was seen in three patients (6.3%, 95%CI = 0.1–17.2) and arterial pulmonary hypertension in one infant. No cases of altered ventricular function, pericardial effusion, or coronary anomalies were found. One additional patient presented with left ventricular hypertrophy without dysfunction which was unrelated to the mother’s gestational diabetes. Two infants had more than one defect.

Of 48 infants with ECHO abnormalities, 13 infants had major structural defects noted, representing 10.8% of 120 infants (95% CI = 5.9% to 17.8%). None had severe structural cardiac defects. All infants in the group with major structural defects had positive PCR results in CSF and/or urine at birth as seen in Table 1. Six of 13 infants in the group with major defects had microcephaly and 5 had abnormal CNS imaging. (Table 1).

**Table 1:** Characteristics of 13 patients with abnormal ECHO findings.

Patient	Sex	Age at ECHO (days)	PCR (m) ^a^	PCR (n) ^b^	Microcephaly	CNS imaging^c^	Gestational age (weeks)	2D-Echo	Rash-trimester
1	Girl	288	Positive	Positive	Yes	Positive	36	PDA ^d^	2nd
2	Girl	220	Positive	Positive	No	Negative	40	PDA	2nd
3	Boy	132	Positive	Positive	No	Negative	40	VSD ^e^	2nd
4	Boy	1	Positive	Positive	Yes	Positive	39	PAH ^f^	1st
5	Girl	178	Positive	Positive	Yes	Positive	37	PDA	1st
6	Boy	2	Positive	Positive	Yes	Positive	40	VSD	1st
7	Girl	96	Positive	NA	Yes	Negative	38	ASD ^g^	2nd
8	Girl	28	Negative	Positive	No	Positive	37	ASD	3rd
9	Girl	2	Positive	NA	Yes	Negative	35	LVH ^h^	2nd
10	Boy	55	Positive	NA	No	Negative	39	ASD	2nd
11	Boy	91	Positive	Positive	No	Negative	39	VSD	2nd
12	Girl	7	Negative	Positive	No	Negative	38	POF^i^, VSD, RSPB ^j^	No rash
13	Boy	NA^k^	Positive	NA	No	NA	37	VSD, POF	No rash

a-Mother´s PCR

b-Neonate´s PCR

c-Central nervous system imaging

d-Patent ductus arteriosus

e-Ventricular septal defect

f-Pulmonar arterial hypertension

g-Atrial septal defect

h-Left ventricular hyperthrophy

i-Patent foramen oval

j-Relative stenosis of pulmonary branches

k-Not available

Major heart defects were 14% more frequent in preterm infants than in term babies, 10% higher in infants whose mothers had a history of rash in the 2nd trimester as compared to other time points or no rash, and 11%. higher in infants with altered CNS imaging tests. Despite this trend, no statistically significant association was identified. No difference in frequency of cardiac defects was found between infants with and without microcephaly (46.1% in the group with major cardiac defects and 42.9% in the group without cardiac defects). Distribution of findings in infants with normal and abnormal echocardiograms are outlined in Table 2.

**Table 2:** Differences between patients with and without major ECHO findings.


		Normal				Abnormal			
		n	%	95%CI		n	%	95%CI	
Sex	Boys	53	49,5%	39,7%	59,4%	6	46,2%	19,2%	74,9%
	Girls	54	50,5%	40,6%	60,3%	7	53,8%	25,1%	80,8%
Gestational age^a^	Pre-term	26	24,3%	16,5%	33,5%	5	38,5%	13,9%	68,4%
	Term	81	75,7%	66,5%	83,5%	8	61,5%	31,6%	86,1%
Rash(trimester) ^a^	No Rash	8	7,5%	3,3%	14,2%	2	15,4%	1,9%	45,5%
	1st	33	30,8%	22,3%	40,5%	3	23,1%	5,0%	53,8%
	2nd	48	44,9%	35,2%	54,8%	7	53,8%	25,1%	80,8%
	3rd	18	16,8%	10,3%	25,3%	1	7,7%	0,2%	36,0%
CNS imaging tests ^a^ ^b^	Negative	73	69,5%	59,8%	78,1%	7	58,3%	27,7%	84,8%
	Positive	32	30,5%	21,9%	40,2%	5	41,7%	15,2%	72,3%

^a^ Variables with at least 10 percentage points at the difference between percentage of abnormal and normal ECHo groups.

^b^ Central nervous system imaging tests

Note: Fisher exact test p-values: Sex = 1.000, Gestacional age = 0.317, Rash = 0.515, CNS Imaging test = 0.559.

## Discussion

Our study found a 10.8% (IC 95% = 5.9% to 17.8%) prevalence of major structural heart defects in infants with a history of intrauterine exposure to ZIKV, a rate considerably higher than that observed in the general population. However, no patients were found to have severe structural heart defects. These results are similar to those of a study from the state of Pernambuco, Brazil, which evaluated infants with congenital Zika syndrome but without laboratory confirmation of intrauterine exposure to ZIKV. That study demonstrated an incidence of 13.5% of major ECHO defects but also found no severe congenital heart defects [11].

The prevalence of major cardiac defects in our study was 10 times higher in ZIKV infants (13 cases in 120 infants) than that observed in the general population. The reported prevalence of congenital heart defects in live born infants in Atlanta and China respectively was 8.1 to 11.1/1,000 [13,14], with a 1.5/1000 prevalence of severe congenital heart defects reported in. In China and in the U.S. left-to-right shunts (VSD, ASD, and PDA) were the most frequent defects, and accounted for half of the congenital heart defects reported in Atlanta [13]. In the American study, muscular VSD was the most common defect while tetralogy of Fallot was the most common cyanotic heart defect (0.47/1,000). In the Chinese study, prevalence of all types of VSD was 3.7/1,000 and tetralogy of Fallot was also the most common cyanotic congenital heart defect. A study in Nigeria reported similar findings [15]. In Brazil, a recent study reported a prevalence of congenital heart disease of 0.5 per 1000 live births across the country. Nevertheless, this study strongly suggests that there is significant under reporting of cases of congenital cardiac diseases in the country [16]. Previous studies have described congenital heart disease rates in Brazil ranging from 1 per 1000 in live borns and 88 per 1000 in stillbirths [17]. Prior literature on the status of cardiac surgery for congenital heart disease in Brazil emphasizes the significant lack of resources for surgical procedures, with an overall country deficit estimated at 80% (i.e., 80% of infants in need of cardiac surgery in the first year of life did not receive them) [16], whereas mortality data following congenital heart surgery from the Heart Institute of the University of Sao Paulo was reported at 8% in 2016 [18]. This underscores the precarious condition in our country for adequate identification and management of serious congenital heart disease. From our data it appears that Zika-affected infants do not require emergent cardiac interventions.

Except for two infants with left ventricular hypertrophy and pulmonary arterial hypertension, we found left-to-right shunts in all infants with major cardiac defects, which is similar to that reported in previous epidemiologic studies of congenital heart disease. [13,14,15].

In our study the finding of PDA in three neonates who were less than 15 days of age was considered physiological, and the presence of a PFO in 29.2% of our subjects should be analyzed within the context of the infants’ age (all subjects were less than 13 months of age). It is known that in postmortem analyses, the incidence of PFO is 27.3%, but taking age into account, a study showed an incidence of 34.3% in the first three decades of life and a progressive decrease in later decades [19]. However, these patients should be followed to assess whether there is any difference in the incidence of closure with age.

There was a trend in our study for major heart defects to be more frequently observed in infants with preterm birth, in those whose mothers had second trimester of pregnancy infection and in infants who had altered CNS imaging studies. Our findings however were not statistically significant, but this could be a function of sample size. We believe these factors merit further investigation in future Zika infant cohorts that have larger sample sizes.

Although our study found a higher prevalence of structural cardiac defects than anticipated, it did not demonstrate any severe structural heart defects in infants with *in utero* ZIKV-exposure. None of the infants needed cardiac procedures or surgery in the first days to months of life. We believe postnatal assessments performed routinely in maternity hospitals in Brazil which entail a newborn physical exam and a newborn oxygen saturation test prior to discharge from the hospital would be sufficient for identification of infants with major structural heart defects. Our findings support the recommendation that fetuses or infants with suspected or confirmed ZIKV exposure in utero should receive the same cardiac follow-up recommended for pregnant women and newborns in general.

Our study is the first to report echocardiogram findings in infants with laboratory confirmed antenatal exposure to ZIKV. Given the seasonality of ZIKV infection, it is difficult to predict whether it will be possible in the future to report on echocardiographic findings of a larger series of infants with laboratory confirmed diagnosis. It would be important however, to be able to replicate these findings in studies evaluating a larger number of subjects.

## Conclusions

A higher frequency of major heart defects was noted in ZIKV exposed infants as compared to the general population. None of these defects, however were considered severe. In light of our findings, we believe recommendations for fetal echocardiograms in pregnant women with confirmed ZIKV infection and performance of postnatal ECHOs in their infants, should follow the same criteria as those established for the general population of mothers and infants. This entails a complete physical exam at birth and measure of oxygen saturation prior to discharge. Recommendations for emergent echocardiographic evaluations in the neonatal period for all Zika exposed infants do not appear to be warranted, as a higher prevalence of severe heart defects was not identified. This recommendation is very relevant to scenarios where performance of fetal and pediatric echocardiograms is not feasible, including most Brazilian municipalities which lack the ability to perform these studies. All patients in the study with positive ECHO findings are being monitored prospectively so that the spontaneous closure or possible complications are readily identified.

## Supporting information

S1 Checklist Strobe checklist. (DOCX)
